# The Putative De-*N*-acetylase DnpA Contributes to Intracellular and Biofilm-Associated Persistence of *Pseudomonas aeruginosa* Exposed to Fluoroquinolones

**DOI:** 10.3389/fmicb.2018.01455

**Published:** 2018-07-10

**Authors:** Shaunak Khandekar, Veerle Liebens, Maarten Fauvart, Paul M. Tulkens, Jan Michiels, Françoise Van Bambeke

**Affiliations:** ^1^Pharmacologie Cellulaire et Moléculaire, Louvain Drug Research Institute, Université catholique de Louvain, Brussels, Belgium; ^2^Centre of Microbial and Plant Genetics, KU Leuven, Leuven, Belgium; ^3^Center for Microbiology, Vlaams Instituut voor Biotechnologie, Leuven, Belgium; ^4^imec, Leuven, Belgium

**Keywords:** de-*N*-acetylase, gyrase, persister, fluoroquinolones, intracellular infection, biofilm, *Pseudomonas aeruginosa*

## Abstract

Persisters are the fraction of antibiotic-exposed bacteria transiently refractory to killing and are recognized as a cause of antibiotic treatment failure. The putative de-*N*-acetylase DnpA increases persister levels in *Pseudomonas aeruginosa* upon exposure to fluoroquinolones in broth. In this study the wild-type PAO1 and its *dnpA* insertion mutant (*dnpA*::Tn) were used in parallel and compared for their capacity to generate persisters in broth (surviving fraction after exposure to high antibiotic concentrations) and their susceptibility to antibiotics in models of intracellular infection of THP-1 monocytes and of biofilms grown in microtiter plates. Multiplication in monocytes was evaluated by fluorescence dilution of GFP (expressed under the control of an inducible promoter) using flow cytometry. Gene expression was measured by quantitative RT-PCR. When exposed to fluoroquinolones (ciprofloxacin or levofloxacin) but not to meropenem or amikacin, the *dnpA*::Tn mutant showed a 3- to 10-fold lower persister fraction in broth. In infected monocytes, fluoroquinolones (but not the other antibiotics) were more effective (difference in E_max_: 1.5 log cfu) against the *dnpA*::Tn mutant than against the wild-type PAO1. Dividing intracellular bacteria were more frequently seen (1.5 to 1.9-fold) with the fluoroquinolone-exposed *dnpA*::Tn mutant than with its parental strain. Fluoroquinolones (but not the other antibiotics) were also 3-fold more potent to prevent biofilm formation by the *dnpA*::Tn mutant than by PAO1 as well as to act upon biofilms (1–3 days of maturity) formed by the mutant than by the parental strain. Fluoroquinolones induced the expression of *gyrA* (4.5–7 fold) and *mexX* (3.6–5.4 fold) in the parental strain but to a lower extent (3–4-fold for *gyrA* and 1.8–2.8-fold for *mexX*, respectively) in the *dnpA*::Tn mutant. Thus, our data show that a *dnpA* insertion mutant of *P. aeruginosa* is more receptive to fluoroquinolone antibacterial effects than its parental strain in infected monocytes or in biofilms. The mechanism of this higher responsiveness could involve a reduced overexpression of the fluoroquinolone target.

## Introduction

Besides plain resistance most often associated with genomic changes, persistence is increasingly recognized as a cause of therapeutic failures. Persisters represent the fraction of antibiotic-treated bacteria that are transiently refractory to antibiotic killing. This phenotype is not genetically inherited and is reversible upon antibiotic removal (Van den Bergh et al., 2017). Persister formation has been mainly studied in *Escherichia coli* or *Salmonella* Typhimurium, in which species it is supposedly mediated by toxin-antitoxin systems and favored by environmental stress that can lead to antitoxin degradation (Fisher et al., 2017). Only few reports have investigated the mechanism(s) of persister formation in *Pseudomonas aeruginosa*. These have highlighted a role for the quorum-sensing determinants *lasI* and *lasR* (Kayama et al., 2009; Moker et al., 2010), the alternative sigma factor RpoN (Viducic et al., 2007), and the stationary-phase regulators RpoS, DskA, RelA, and SpoT (Murakami et al., 2005; Viducic et al., 2006). A high-throughput screening of *P. aeruginosa* mutant libraries in PA14 and PAO1 identified *dnpA* as another gene involved in persistence (De Groote et al., 2009; Liebens et al., 2014, 2016). *dnpA* is part of the LPS biosynthesis cluster. Based on its deduced amino acid sequence, DnpA is a member of the LmbE-like superfamily of metalloenzymes that are defined by a phosphatidylinositol glycan anchor biosynthesis class L domain (Viars et al., 2014). *dnpA* encodes a putative de-*N*-acetylase predicted to act on the *N*-acetylglucosamine moiety of a still unknown substrate. Previous work (Liebens et al., 2014) showed that *dnpA* expression increases persister levels upon exposure to ofloxacin in broth while its deletion generates the opposite effect. Preliminary data suggest that LPS synthesis is unaffected in the deletion mutant.

Persisters have been associated with transient dormant lifestyles, notably survival in eukaryotic cells and biofilms (Conlon et al., 2015; Fisher et al., 2017), both of which have been described in *P. aeruginosa. P. aeruginosa* can indeed infect eukaryotic cells and survive intracellularly for extended periods of time (Mathy-Hartert et al., 1996; Kierbel et al., 2005; Schmiedl et al., 2010; Buyck et al., 2013). It also forms biofilms, particularly in the lungs of patients suffering from cystic fibrosis (Costerton, 2001; Bjarnsholt et al., 2009; Høiby et al., 2010). The *P. aeruginosa* biofilm matrix contains three major polysaccharide components, namely alginate, Pel, and Psl (Ryder et al., 2007). *O-*acetylation of alginate by alginate acetylases is important for biofilm architecture (Nivens et al., 2001).

The aim of the present study was to assess the possible role of DnpA in the intraphagocytic and biofilm environments of *P. aeruginosa* when exposed to representative antipseudomonal antibiotics. We show that *dnpA* inactivation increases the efficacy of fluoroquinolones against intracellular *P. aeruginosa* and also makes them more potent against biofilms formed by these bacteria. In parallel, we noticed a lower induction of *gyrA* expression (encoding a subunit of DNA gyrase, the fluoroquinolone target) in the mutant compared to the parental strain when exposed to fluoroquinolones. Although a possible relation between this change and the putative enzymatic function of the DnpA enzyme remains to be established, our study throws new light upon the possible role of DnpA in the impaired response of *P. aeruginosa* to fluoroquinolones in models of persistent infections.

## Materials and Methods

### Bacterial Strains, Primers and Plasmids

Bacterial strains are shown in **Table 1**. *E. coli* and *P. aeruginosa* were grown in Luria-Bertani (LB) broth and Mueller-Hinton broth (MHB) or MHB cation-adjusted (MHB-CA), respectively. Plasmids and primers are shown in **Tables 1, 2**, respectively. The *dnpA*::Tn mutant contains a transposon insertion at ORF position 1248 nt out of 1418 nt. The possible synthesis of a truncated DnpA protein of 416 aa instead of 473, i.e., with 57 aa missing, can, therefore, not be ruled out but this protein is unlikely to be functional. The transcription units in *P. aeruginosa* have been experimentally determined (Wurtzel et al., 2012), demonstrating that *dnpA* is part of the transcription unit PA5005-*wpaH*/PA5004-PA5003-*dnpA*/PA5002. Consequently, no polar effects on the expression of the downstream gene PA5001 (*ssg*) are expected. We previously demonstrated that pME6032-*dnpA* in *P. aeruginosa* results in 18-fold overexpression of *dnpA* mRNA when compared to the wild-type strain (Liebens et al., 2014). Given the generally close link between mRNA and protein expression levels in bacteria, it can reasonably be assumed that *dnpA* mRNA overexpression will also result in increased DnpA protein levels.

**Table 1:** Bacterial strains and plasmids used in the study.

Strains	Description	Reference
*Pseudomonas aeruginosa*		
PAO1	Wild-type	Holloway, 1955
*dnpA*::Tn	PAO1 with an insertion in *dnpA* gene (PA5002), Tc^r^	Jacobs et al., 2003
*PAO1(dnpA)*	transformed by pME6032 plasmid withPA14 *dnpA* gene (PA5002), Tc^r^	Liebens et al., 2014
PAO1(GFP-iptg)	PAO1 harboring pBBR5-GFP construct	This study
*dnpA*::Tn (GFP-iptg)	*dnpA*::Tn harboring pBBR5-GFP construct	This study
PAO1(GFP-ara)	PAO1 harboring pHERD-GFP construct	This study
*dnpA*::Tn (GFP-ara)	*dnpA*::Tn harboring pHERD(Amp)-GFP construct	This study
*Escherichia coli*		
DH5α	supE44 D*lac*U169 (F80 *lac*ZDM15) *hsd*R17 *rec*A1 *end*A1 *gyr*A96 *thi*-1 *rel*A1	Sambrook and Russell, 2001

**Plasmids**		

pHERD26T	pUCP26 P_lac_ replaced with 2.4-kb AdhI-EcoRI fragment of *ara*C-P_BAD_ cassette and *ori*T, Tc^r^	Qiu et al., 2008
pHERD(Amp)	PCR amplified beta-lactamase (*bla*) cassette inserted into pHERD26T vector using HindIII, Ap^r^	This study
pMP4655	*gfp* containing construct, constructed by ligation of pVS1 derived shuttle vector pME6010 and broad host range vector pBBR1MCS-5, *gfp* under control of P_lac_, Tc^r^	Bloemberg et al., 2000
pBBR5-GFP	*gfp* containing broad host range vector pBBR1MCS-5, formed after digestion of pMP4655 using BglII, Gm^r^	This study
pHERD-GFP	*gfp* containing pHERD26T construct, cloned from pBBR5-GFP using XbaI-KpnI, under control of P_BAD_, Tc^r^	This study
pHERD(Amp)-GFP	*bla* containing pHERD-GFP construct, Ap^r^	This study

*Tc, tetracycline; Ap, β-lactamase/ampicillin; Gm, gentamicin; ^*r*^, resistance.*

**Table 2:** Primers used in the study.

Name	Sequence
amp_fw	CCC*AAGCTT*TCCGCTCATGAGACAATAACC
amp_rv	CCC*AAGCTT*TTGGTCTGACAGTTACCAATGC
gyrA_fw	GAGCTGCCGTACCAGTTGAA
gyrA_rv	GGGTCTGGGCATAGAGGTTG
gyrB_fw	GAGTACATGACCCAGTCGGC
gyrB_rv	ATGAAGTGCTCGGTCAGCTC
rspL_fw	CGGCACTGCGTAAGGTATGC
rspL_rv	CGTACTTCGAACGACCCTGCT
mexA_fw	CGACCAGGCCGTGAGCAAGCAGC
mexA_rv	GGAGACCTTCGCCGCGTTGTCGC
mexX_fw	TGAAGGCGGCCCTGGACATCAGC
mexX_rv	GATCTGCTCGACGCGGGTCAGCG

*^a^Nucleotides in italics represent HindIII restriction endonuclease recognition site.*

### Transformation of *Pseudomonas*

Green fluorescent protein (GFP) was used as a reporter under the control of promoters P_lac_ or P_BAD_. In case of *P. aeruginosa*, even though the expression from P_lac_ was constitutive, 1 mM IPTG was added (this addition is not expected to affect GFP expression). pMP4655 construct (Bloemberg et al., 2000) was isolated from *E. coli* DH5α and digested using restriction endonuclease BglII. The resulting two fragments were two complete vectors, namely pME6010 (Heeb et al., 2000) and pBBR1MCS-5 encoding GFP. The fragments were thus religated (T4 DNA Ligase, Thermo Scientific) and the pBBR5(GFP) construct was selected on LB agar containing gentamicin (5 mg/L). For P_lac_, this PBBR5(GFP) construct was transformed into PAO1 and its *dnpA*::Tn deletion by electroporation (Choi et al., 2006). For P_BAD_, the GFP-encoding fragment was then cleaved using restriction enzymes XbaI and KpnI, purified via gel extraction (QIAquick gel extraction kit, Qiagen, Hilden, Germany), and ligated into pHERD26T (Qiu et al., 2008). The construct was transformed into PAO1 by electroporation. For *dnpA*::Tn, an additional PCR-amplified (**Table 2**) ampicillin resistance cassette was cloned into this construct using restriction enzyme HindIII, before transforming into *dnpA*::Tn. Arabinose (0.2%) was used for induction of GFP expression in *dnpA*::Tn (GFP-ara) used for flow cytometry analyses. Antibiotics were added at the following concentrations to maintain plasmids: tetracycline: 20 mg/L for *E. coli*, 100 mg/L for *P. aeruginosa*; ampicillin: 50 mg/L for *E. coli*, carbenicillin: 250 mg/L for *P. aeruginosa*; gentamicin: 5 mg/L for *E. coli.*

### Susceptibility Testing

Minimum inhibitory concentrations (MICs) were determined by microdilution in MHB-CA following Clinical and Laboratory Standards Institute (2017) recommendations and using *P. aeruginosa* ATCC27853 as quality control.

### Persister Fraction Determination

The persistence assay was performed as described previously with minor modifications (De Groote et al., 2009). Briefly, bacteria (PAO1, *dnpA*::Tn) were allowed to grow up to reach a stationary phase after two sub-cultures in MHB. They were then exposed to the antibiotic under study (at a concentration corresponding to 50 times its MIC [see **Table 3** for MIC values]) or to the vehicle (sterile water [control]) in MHB for 5 h at 37°C with shaking at 250 rpm. At the end of this incubation period, the number of cfu/mL was determined by plate counts. The persister fraction was calculated as the ratio between the number of surviving bacteria after antibiotic treatment compared to that measured in control conditions for *dnpA*::Tn *vs*. PAO1.

**Table 3:** Pertinent regression parameters of concentration-response curves for intraphagocytic activities of antibiotics against *P. aeruginosa* strains^a^.

Antibiotic	Strains					
	PAO1			*dnpA*::Tn		
	E_max_^b^	MIC^c^	C_s_^d^	E_max_	MIC	C_s_
Ciprofloxacin	-2.34 ± 0.14 (A)^e^	0.25	0.34/1.36	-3.94 ± 0.19 (B)	0.25	0.27/1.08
Levofloxacin	-3.76 ± 0.41 (A)	1	3.18/3.18	-5.15 ± 0.29 (B)	1	1.91/1.91
Meropenem	-0.69 ± 0.28 (A)	2	5/2.50	-1.31 ± 0.25 (A)	2	3.4/1.70
Amikacin	-1.19 ± 0.40 (A)	4	23.9/5.98	-1.31 ± 0.65 (A)	4	18.9/4.73

*^a^Calculated based on the concentration-response curves (sigmoidal dose-response curves with Hill slope = 1) of experiments shown in **Figure 2**. ^b^E_max_: maximal decrease in inoculum (in log_10_ units) compared to the post-phagocytosis inoculum as extrapolated from the Hill equation of the concentration-response curve for an infinitely large antibiotic concentration. ^c^MIC, minimal inhibitory concentration in broth (mg/L). _d_C_s_, static concentration, i.e., the extracellular concentration (expressed in two different units, namely: mg.L^-1^/x MIC) resulting in no apparent bacterial growth (number of cfu identical to the post-phagocytosis inoculum). _e_Statistical analysis (1 way ANOVA with Tukey post-hoc test): Values with different letters are significantly different from one another when comparing each antibiotic against PAO1 vs. dnpA::Tn.*

### Gene Expression

At the end of the persister fraction determination assay, bacteria were harvested by centrifugation and total RNA was isolated using the InviTrap Spin Universal RNA Mini Kit (Stratec Molecular GmbH, Berlin, Germany). cDNA was obtained by reverse transcription of purified RNA using Transcriptor First Strand cDNA Synthesis Kit (Roche, Basel, Switzerland) and used to evaluate the expression of *gyrA, gyrB, mexA*, and *mexX* by quantitative reverse transcriptase (qRT-PCR) in an iQ cycler^TM^ Real-Time PCR Detection System (Bio-Rad, Hercules, CA, United States), with *rpsL* as housekeeping gene and iQ SYBR Green Supermix as detection system (Bio-Rad). The PCR conditions were: 1 cycle for 3 min at 95°C, followed by 40 cycles of 10 s at 95°C, 30 s at 58°C, and 30 s at 72°C, with the primers given in **Table 2**. A melting curve was run at the end of the PCR cycles to check for the presence of a unique PCR reaction product. The analysis was performed by the comparative C_T_ method, with the fold-change calculated as 2^-ΔΔCT^. In this equation, ΔΔC_T_ = (ΔC_T_ [antibiotic-exposed]-ΔC_T_ [no antibiotic]), and ΔC_T_ = C_T_ [gene of interest] – C_T_ [*rspL* as housekeeping gene] (Livak and Schmittgen, 2001). Data were expressed as means and range of values (Applied-Biosystems, 2008).

### Intracellular Infection of THP-1 Monocytes

Human THP-1 cells were cultivated in RPMI-1640 medium supplemented with 10% fetal calf serum (FCS). Intracellular infection was performed exactly as previously described (Buyck et al., 2013), using PAO1, the *dnpA*::Tn insertion mutant, and PAO1(dnpA) overexpressing *dnpA*. Infected cells were incubated with antibiotics (extracellular concentrations ranging from 0.01 to 200 mg/L) for 24 h, collected in distilled water (with cell lysis checked by visual inspection in optical microscopy), and the residual inoculum determined by cfu counting normalized to total sample protein content (DC Protein Assay; Bio-Rad). Activity was expressed as change from the initial inoculum after 24 h, and the data were used for fitting a concentration-response curve (Hill equation), and calculating the E_max_ and C_static_ (C_s_) pharmacodynamic parameters (Buyck et al., 2016).

### Flow Cytometry Analysis

We followed a previously published method (Roostalu et al., 2008) with slight modifications and using arabinose-inducible reporters. For extracellular bacteria, we followed the protocol of the persister assay. Bacteria [PAO1(GFP-ara) or *dnpA*::Tn (GFP-ara)], were collected by centrifugation and at least 10,000 events were counted. For intraphagocytic bacteria, we applied the protocol of intracellular infection but added 0.2% arabinose in all the steps until the post-phagocytosis washing with PBS (which also served to remove residual arabinose). For time-zero sample, 1 mL infected THP-1 monocytes were lysed with sterile water, centrifuged, resuspended in 100 μL PBS and immediately analyzed using a flow cytometer (FacsVerse; Becton Dickinson and Company, Franklin Lakes, NJ, United States) with laser beam and band pass filter set at 488 nm and 527/32 nm, respectively. For 24 h samples, infected monocytes were incubated with antibiotics at 2x or 50x MIC, harvested and analyzed. 2,000–2,500 events per sample were counted to avoid increase in cell debris and false positive events. The results were analyzed by FlowJo software (FlowJo LLC, Ashland, OR, United States).

### Biofilm Assay

A bacterial suspension (10^8^ cfu/mL) of PAO1, the *dnpA*::Tn *insertion* mutant and PAO1 (dnpA) was diluted 100x in MHB-CA medium before dispensing 200 μL into 96-well plates (VWR, Radnor, PA, United States). The medium was replaced with fresh medium every day for up to 4 days so as to generate a mature biofilm. Antibiotics were added at extracellular concentrations ranging from 0.01 to 200 mg/L and incubated at 37°C for 24 h. Bacterial viability was quantified using fluorescein di-acetate (FDA, Sigma–Aldrich, St Louis, MO, United States), which is reduced to fluorescein by viable bacteria (Peeters et al., 2008). At the end of the incubation period, the medium was removed and the wells washed with PBS. Biofilms were incubated 1 h with 10 mg/L FDA at 37°C in the dark. Fluorescein fluorescence was measured (excitation/emission wavelengths: 494/518 nm) in a SPECTRAmax Spectrofluorometer (Molecular Devices, Sunnyvale, CA, United States). Cell viability was expressed as percentage of fluorescence signal for antibiotic-exposed *vs.* control biofilms. Biomass was measured by crystal violet staining (Peeters et al., 2008). Briefly, the biofilm was washed once with PBS and fixed with 100% methanol for 20 min. After removing the methanol and air-drying the biofilm, 200 μL of 10% crystal violet was added for 20 min, after which the biofilm was gently washed with water to remove unbound dye. Bound crystal violet was solubilized with 200 μL of 66% (v/v) acetic acid. After 1 h incubation at room temperature, the absorbance of the solution in each well was measured at 590 nm, and the value used as a measure of the biomass. In preliminary experiments, we checked for the linearity of the signal measured as a function of crystal violet concentration in the range of absorbance measured in biofilms; dilutions were applied if needed. Change in biomass upon exposure to antibiotics was expressed as the percentage of the value measured for biofilms that had not been exposed to antibiotics.

## Results

### Extracellular Persistence Assay

**FIGURE 1:**
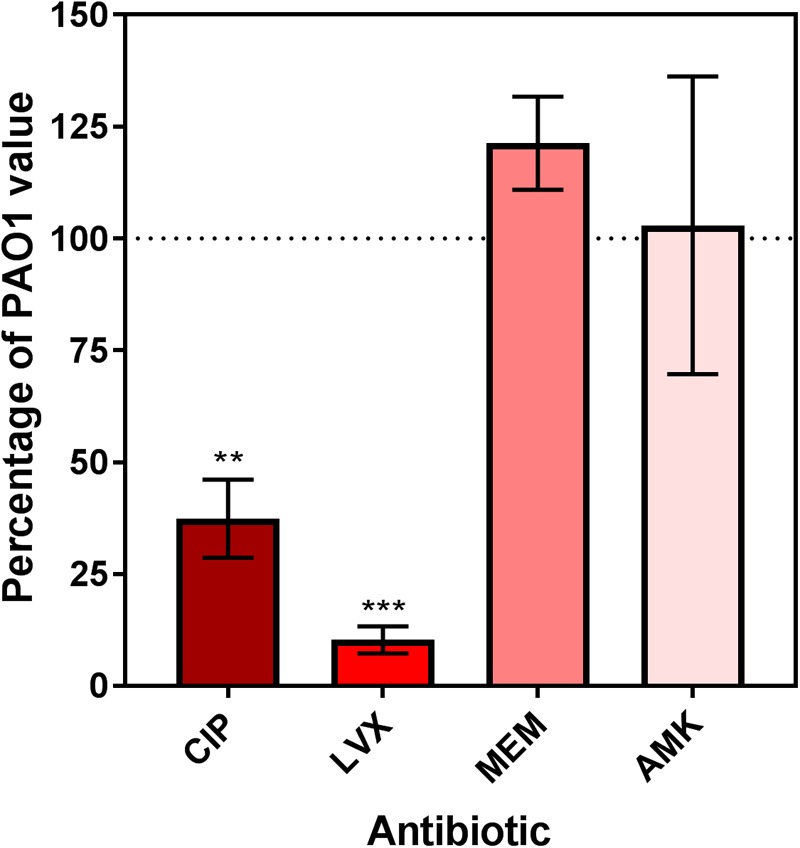
Relative persister fraction for extracellular *dnpA*::Tn expressed as percentage of the value measured for PAO1. Bacteria were exposed during 5 h to antibiotics at a concentration of 50x MIC. Persister fraction was determined as the number of cfu for bacteria exposed to the drug *vs.* the corresponding untreated control. The horizontal dotted line corresponds to the reference value (PAO1). Data are means ± SEM of at least 3 experiments performed in triplicates. Statistical analysis (multiple *t*-test) comparing *dnpA*::Tn to PAO1: ^∗∗^*p* < 0.01 and ^∗∗∗^*p* < 0.001. CIP, ciprofloxacin; LVX, levofloxacin, MEM, meropenem, AMK, amikacin.

Previous work has shown that the inactivation of *dnpA* significantly lowers the persister fraction in PAO1 when exposed to low concentrations of ofloxacin, a fluoroquinolone antibiotic (De Groote et al., 2009). Here, we extended our observations to another fluoroquinolone (ciprofloxacin) and to the active isomer of ofloxacin (levofloxacin) as well as to one typical representative of the β-lactams (meropenem) and of the aminoglycosides (amikacin). We also used higher antibiotic concentrations (50x MIC). A drastic reduction in the persister fraction was observed for *dnpA*::Tn as compared to PAO1 after exposure to both fluoroquinolones, but not to meropenem or to amikacin (**Figure 1**).

### Intracellular Infection and Antibiotic Intracellular Activity

**FIGURE 2:**
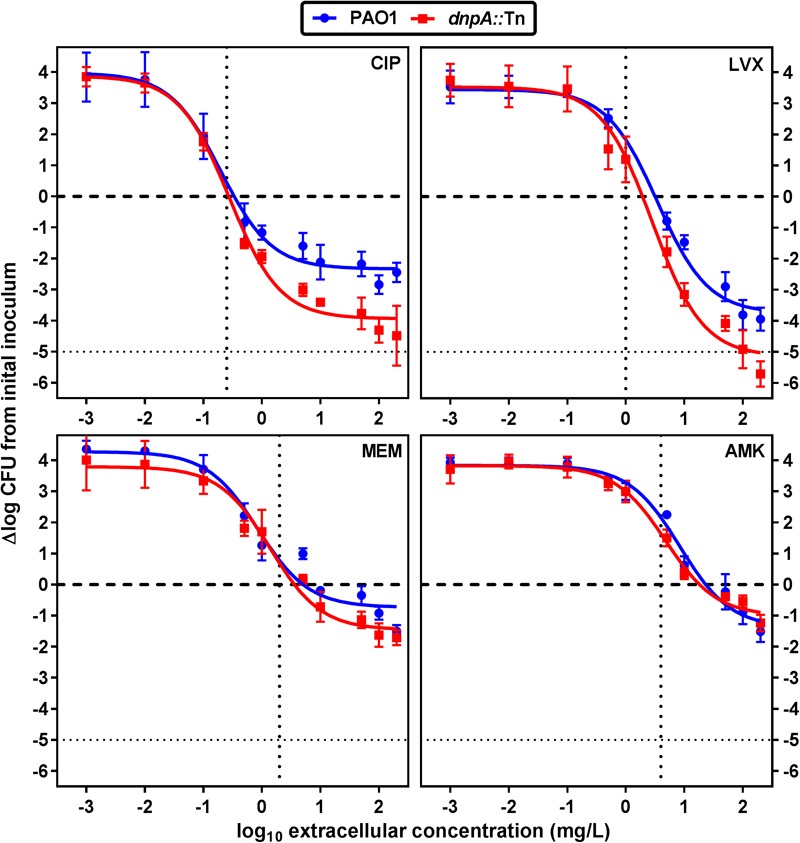
Concentration-response curves of antibiotics against intracellular *P. aeruginosa*. The graphs show the changes in the number of cfu/mg of cell protein in THP-1 cells after 24 h of incubation with increasing extracellular antibiotic concentrations. Values are the mean ± SEM of at least 3 experiments performed in triplicates. Vertical dotted lines indicate MIC value in broth. The tick horizontal dotted line indicates a bacteriostatic effect and the thin horizontal dotted line, the limit of detection. CIP, ciprofloxacin; LVX, levofloxacin, MEM, meropenem, AMK, amikacin. See **Table 3** for pharmacodynamic parameters and statistical analysis.

We first compared the activity of both fluoroquinolones (ciprofloxacin and levofloxacin) and of meropenem and amikacin against the *dnpA*::Tn and PAO1 strains in infected human THP-1 monocytes after 24 h of incubation and using a wide range of extracellular concentrations to obtain full concentration-response curves. We have previously measured the accumulation of antibiotics representative of the classes used here in THP-1 cells, with the following values observed at or close to equilibrium, or at fixed time point: aminoglycosides (gentamicin): 4 x (slow accumulation), fluoroquinolones (fast accumulation): 5 x (ciprofloxacin); 7 x (levofloxacin) and β-lactams (meropenem): 1 x at equilibrium (Carryn et al., 2002; Barcia-Macay et al., 2006). Their intracellular activity is illustrated in a graphical format in **Figure 2** and the corresponding key pharmacodynamic parameters (as calculated from the corresponding Hill’s functions fitted to the data) are shown in **Table 3**. As previously described (Buyck et al., 2013), meropenem and amikacin were poorly effective against intraphagocytic *P. aeruginosa*, with maximal relative activities (E_max_) not exceeding about 1 to 2 log_10_ cfu decrease compared to the original post-phagocytosis inoculum. Conversely, and as also reported earlier, the fluoroquinolones were more efficacious. Most strikingly, the maximal relative efficacies (E_max_) of both fluoroquinolones was significantly larger (more negative E_max_) against *dnpA*::Tn than PAO1 while their relative potencies (evaluated by the value of the apparent static concentration [C_s_]) remained essentially unchanged. Of note, the PAO1(dnpA) construct that overexpresses *dnpA* behaved as the wild-type strain (Supplementary Figure 1 for data with ciprofloxacin). Additional control experiments included the demonstration that (a) the remaining intraphagocytic bacteria were not resistant mutants (same MIC for randomly picked colonies collected from cells exposed to 50x MIC compared to the initial strain) and (b) both GFP-expressing strains were localized in LysoTracker Red-positive acidic bodies as previously described (Buyck et al., 2013) (Supplementary Figure 2).

**FIGURE 3:**
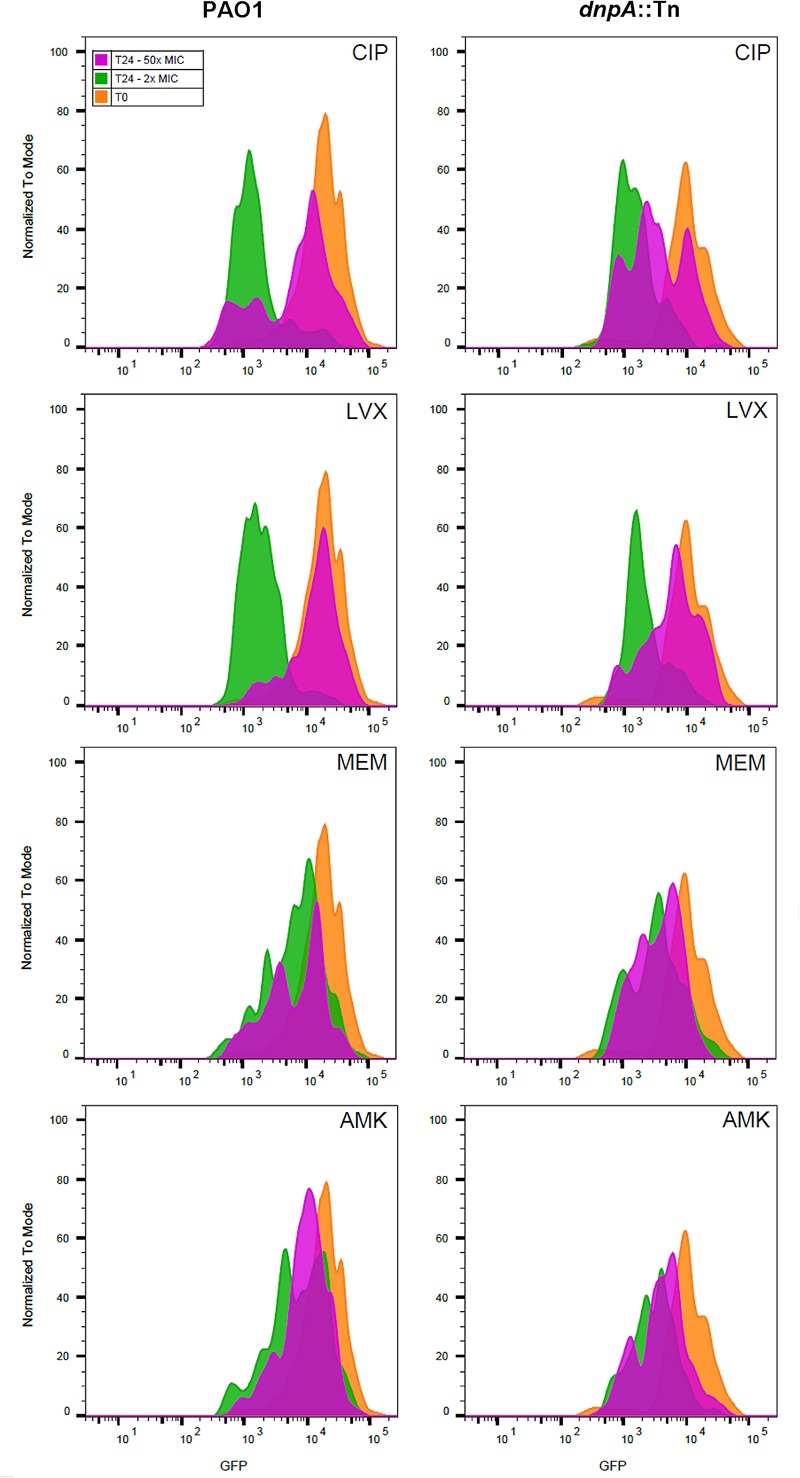
Fluorescence dilution experiment performed on intraphagocytic *P. aeruginosa* strains expressing GFP. The histograms show the number of events (normalized to mode) as a function of the intensity in GFP signal. Infected monocytes were harvested after phagocytosis (T0, orange histogram) or exposed during 24 h (T24) to 2x (green histograms), or 50x (pink histograms) MIC of antibiotics. Bacteria were harvested after lysing the THP-1 monocytes with H_2_O. At least 2,000 events were measured for each sample. CIP, ciprofloxacin; LVX, levofloxacin, MEM, meropenem, AMK, amikacin.

**FIGURE 4:**
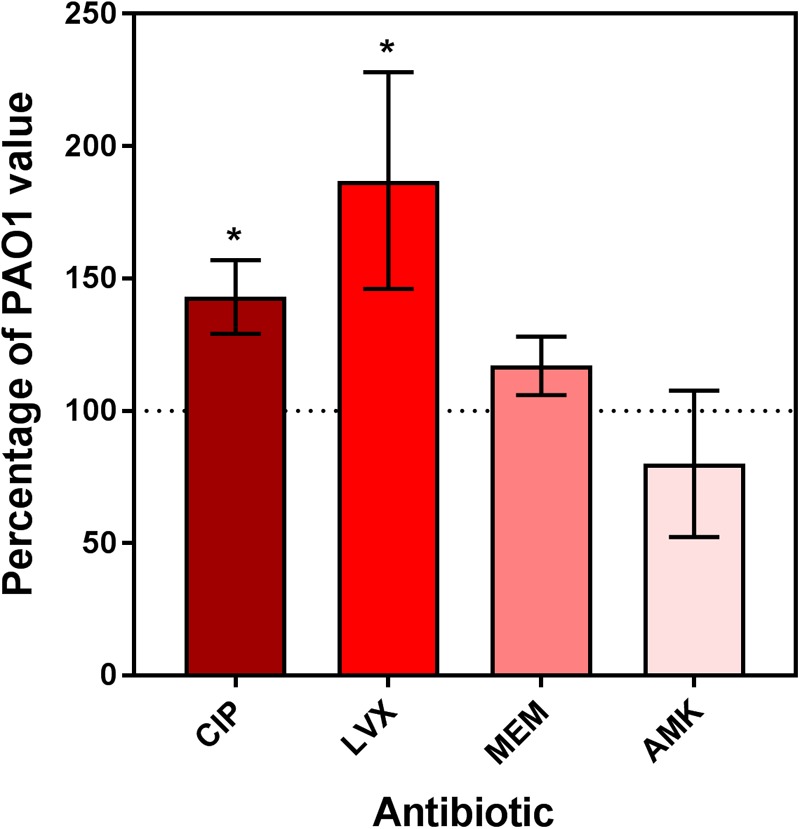
Relative fraction of intraphagocytic *dnpA* insertion mutant as compared to PAO1 showing less than 10^3.5^ of GFP fluorescence after 24 h of incubation with antibiotics at a concentration corresponding to 50x MIC. The horizontal dotted line corresponds to the reference value (PAO1). Data are the mean ± SEM of at least 3 experiments performed in triplicates. Statistical analysis (multiple *t*-test) comparing *dnpA*::Tn to PAO1: ^∗^*p* < 0.05 and ^∗∗^*p* < 0.01. CIP, ciprofloxacin; LVX, levofloxacin, MEM, meropenem, AMK, amikacin.

As the previous results obtained with extracellular bacteria (broth) indicated that *dnpA*::Tn formed less persisters than PAO1 when exposed to fluoroquinolones, we aimed at determining whether the same phenomenon could explain the difference in maximal relative efficacies (E_max_) observed here with the intraphagocytic bacteria. Flow cytometry has been proposed as a tool for visualizing bacterial growth. To this effect, fluorescent proteins are expressed under the control of an inducible promoter, after which the bacteria are transferred to an inducer-free medium. Therefore the fluorescence signal will decrease if bacteria divide (due to the dilution of the tracer) but will remain unchanged if bacteria do not divide (Roostalu et al., 2008). We first validated the approach using arabinose-induced overnight bacterial cultures of PAO1 transformed with the GFP-encoding construct [PAO1(GFP-ara)] and exposed to arabinose for induction of GFP. After removing the inducer, we followed the fluorescence signal intensity periodically over 8 h and found, as expected, a gradual reduction in its intensity as detected by flow cytometric analysis (Supplementary Figure 3). THP-1 cells were then infected with PAO1(GFP-ara) or *dnpA*::Tn (GFP-ara), after which the fluorescence signal was measured immediately (0 h) and after 24 h of incubation of the infected cells with antibiotics at a low (2x MIC) or a high (50x MIC) concentrations. **Figure 3** shows the frequency distribution of fluorescence associated with approximately 2,000 events (i.e., ∼2,000 intracellular bacteria). At time 0, the range of fluorescence values recorded for the *dnpA*::Tn mutant was slightly broader than for the parental strain. No or minor dilution of the fluorescence signal was observed for either strains when THP-1 cells were incubated in the presence of meropenem or amikacin at 2x or 50x MIC, indicating that most of the residual intraphagocytic inoculum that survived the antibiotic treatment was not dividing in these conditions. In contrast, a clear decrease in the signal intensity was noticed for both strains when THP-1 cells were exposed to fluoroquinolones at 2x MIC or at 50x MIC, but only for a part of the population in the latter case, suggesting that part of the residual intraphagocytic inoculum remains in a metabolic state capable of multiplying in these conditions. A quantitative analysis of these data is presented in **Figure 4**, which shows the relative fraction of intraphagocytic *dnpA*::Tn mutant *vs.* PAO1, with a GFP fluorescence level lower than 10^3.5^ after 24 h of incubation with antibiotics at a concentration corresponding to 50x MIC [this threshold value of fluorescence was selected to correspond to a 32-fold multiplication rate of bacteria (see Supplementary Figure 3), corresponding approximately to the 1.5 log cfu difference observed in the E_max_ of fluoroquinolones against these two strains in the intracellular infection model]. Thus, the graph shows that the fraction of surviving bacteria still capable of dividing is higher for intracellular *dnpA*::Tn (GFP) than for PAO1 after 24 h incubation with 50x MIC of fluoroquinolones but not of other antibiotics.

### Biofilms and Antibiotic Activity

**FIGURE 5:**
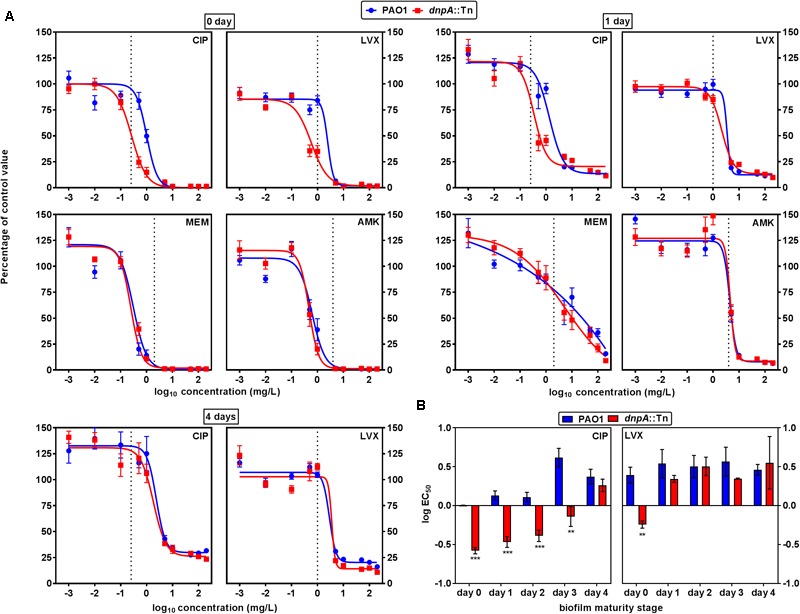
**(A)** Concentration-response curves of the activity of antibiotics against cell viability in 0, 1, or 4 days-old biofilms of *P. aeruginosa* PAO1 (blue) or *dnpA*::Tn (red). The ordinate shows the percentage of residual fluorescein fluorescence signal as compared to control biofilms. The vertical dotted line indicates MIC value in broth. CIP, ciprofloxacin; LVX, levofloxacin; MEM, meropenem; AMK, amikacin. **(B)** Comparison of the logEC_50_ (calculated from the Hill equation of the concentration-response curve presented in **(A)** or of similar experiments at days 2 or 3) as a measure of the relative potency of fluoroquinolones against bacterial viability in biofilms. Values are the means ± SEM of minimum 3 experiments performed in triplicates. Statistical analysis (multiple *t*-test) comparing *dnpA*::Tn to PAO1: ^∗∗^*p* < 0.01 and ^∗∗∗^*p* < 0.001.

We then examined the activity of antibiotics against PAO1 and *dnpA*::Tn in a static model of biofilms grown in 96-well plates for up to 4 days. As shown in Supplementary Figure 4, there was no difference in the kinetics of biofilm growth between the two strains. Antibiotics were added to biofilms with different maturity stages, using the same wide range of concentrations as in our studies of intracellular infection, and their activity evaluated after 24 h with respect to change in viability (**Figure 5**) and of biomass (Supplementary Figure 5). Data at day 0 illustrate the effect of antibiotics on biofilm formation. While biofilm formation was prevented by meropenem and amikacin at concentrations close to their MIC, higher concentrations were needed for the fluoroquinolones. No difference in activity of meropenem or amikacin was observed when comparing their effect on biofilm formation by *dnpA*::Tn and PAO1, while both fluoroquinolones were more potent against *dnpA*::Tn than against PAO1, regarding viability and biomass. Against preformed biofilms, levofloxacin, meropenem and amikacin showed the same profile of activity (as illustrated for day 1 in the top right panel of the figure) while ciprofloxacin remained more potent against the *dnpA*::Tn mutant than against PAO1. This difference in ciprofloxacin potency against *dnpA*::Tn *vs.* PAO1 was statistically different for biofilms aged 1–3 days but vanished at day 4 (**Figure 5A**, bottom left graphs and **Figure 5B**). No difference was observed between the two strains when examining the activity of antibiotics against biomass on preformed biofilms. Again, the PAO1(dnpA) construct behaved as the wild-type strain (Supplementary Figure 1 for data with ciprofloxacin). Biofilms were also observed by confocal microscopy, using GFP-producing bacteria (see Supplementary Figure 6), illustrating a reduction in the viability signal in the presence of ciprofloxacin (2x MIC) which was slightly larger for the biofilm from the *dnpA*::Tn mutant than for that from the parental PAO1 strain. Since changes in matrix composition could have affected the antibiotic activity, we compared the content in exopolysaccharides, proteins, and DNA of the biofilms produced by both strains. There was only a (non-significant) trend to a lower content of alginate and DNA in the matrix of the biofilm produced by the *dnpA*::Tn mutant as compared to its parental PAO1 strain (Supplementary Figure 7).

### Gene Expression

A striking observation throughout this work was that only fluoroquinolones showed an increased activity against *dnpA*::Tn *vs.* PAO1 in the different studied models. Fluoroquinolones act as direct inhibitors of DNA synthesis by forming ternary complexes with the double strand DNA and the GyrA or ParC subunits of the DNA gyrase and topoisomerase IV, respectively. We therefore measured the expression of *gyrA* and *gyrB* in bacteria that had survived exposure to antibiotics in the persister assay in broth, in parallel with the expression of genes encoding efflux pumps (*mexA* and *mexX*). While fluoroquinolones induced the expression of *gyrA* and *mexX*, and to a lower extent, of *gyrB* and *mexA*, the induction of *gyrA* and *mexX* was lower in *dnpA*::Tn than in PAO1 (**Table 4**). In addition, amikacin reduced the expression of *mexX* and meropenem increased that of *mexA* in *dnpA*::Tn.

**Table 4:** Relative mRNA^a^ expression levels of *gyrA, gyrB, mexA*, and *mexX* genes after exposure of PAO1 and its *dnpA* insertion mutant to different antibiotics.

Antibiotic	*gyrA*		*gyrB*		*mexA*		*mexX*	
	PAO1	*dnpA*::Tn	PAO1	*dnpA*::Tn	PAO1	*dnpA*::Tn	PAO1	*dnpA*::Tn
Ciprofloxacin	4.2^b^	2.8	1.8	1.4	2.8	2.0	3.7	2.8
	(2.5 to 7.1)	(2.0 to 4.0)	(-1.3 to 3.9)	(0.7 to 2.8)	(1.9 to 4.1)	(1.3 to 3.1)	(3.4 to 4.1)	(1.9 to 4.1)
Levofloxacin	6.0	4.0	2.2	1.9	2.3	2.3	5.4	1.6
	(4.2 to 8.5)	(3.9 to 4.2)	(1.4 to 3.4)	(1.5 to 2.3)	(1.2 to 4.4)	(1.8 to 2.9)	(4.0 to 7.3)	(1.5 to 1.7)
Meropenem	1.1	-1.5	-1.1	-1.9	-1.5	1.7	-1.02	-2.2
	(-1.6 to 2.2)	(-5.0 to 2.2)	(-1.6 to 1.4)	(-5.6 to 1.6)	(-2.3 to 1.1)	(1.4 to 2.0)	(-1.2 to 1.2)	(-4.4 to -1.9)
Amikacin	1.8	1.6	1.1	-1.1	1.6	1.8	1.5	-3.2
	(1.2 to 2.6)	(-1.0 to 2.8)	(-1.7 to 1.9)	(-1.8 to 1.6)	(-1.1 to 3.0)	(1.2 to 2.7)	(1.2 to 1.9)	(-5.4 to -1.9)

*^a^RNA was harvested from the surviving bacteria after 5 h of incubation with 50x MIC antibiotic in broth. _b_calculated as 2^-ΔΔ*CT*^, with ΔΔ*C*_*T*_ = (Δ*C*_*T*_ [antibiotic-exposed]-Δ*C*_*T*_ [no antibiotic]), and ΔC_*T*_ = *C*_*T*_ [gene of interest] – *C*_*T*_ [*rspL*]. Data are means (with ranges between brackets) of minimum 3 experiments performed in triplicates.*

## Discussion

This study is, to the best of our knowledge, the first one to document the possible role of *dnpA* (encoding a putative de-*N*-acetylase) in the poor response of *P. aeruginosa* to fluoroquinolones in models of persistent infections. *dnpA* insertion mutants were previously shown to generate less persisters in broth when exposed to ofloxacin (De Groote et al., 2009; Liebens et al., 2014). Here, we extend this observation to ciprofloxacin and confirm it for levofloxacin (the pure active isomer of ofloxacin [racemic mixture]). Moreover, we show that both ciprofloxacin and levofloxacin are more effective against intraphagocytic bacteria and more potent on young biofilms when tested against the *dnpA* insertion mutant than its parental isogenic strain PAO1.

Focusing first on intraphagocytic activity, we reported in details in previous publications that the maximal relative efficacy (E_max_) of fluoroquinolones against phagocytized *P. aeruginosa* is considerably lower (less negative) than against their extracellular forms, but could not offer a mechanistic explanation. In planktonic cultures, it is well-known that dormant or non-growing bacteria are more tolerant to antibiotics (Gilbert et al., 1990; Balaban et al., 2004). For a facultative intracellular bacterium like *Salmonella* Typhimurium, it has been shown that part of the intracellular inoculum ceases to grow, while remaining metabolically active, subsequently adopting a persister-like phenotype (Helaine et al., 2010). Our FACS analyses indicate that, when exposed to antibiotics, intracellular *P. aeruginosa* also tends to decrease its multiplication rate, suggesting a partial switch to a persister phenotype. Most conspicuously, this decrease was lower for the *dnpA* insertion mutant when infected THP-1 monocytes were exposed to fluoroquinolones, consistent with a lower intracellular persister character for the mutant in these conditions, and explaining why the intracellular maximal relative activity (E_max_) of fluoroquinolones is increased (more negative values).

Moving now to biofilms, recalcitrance to antibiotic exposure of the encased bacteria results from a conjunction of factors that include a reduced growth rate, binding of the drugs to biofilm matrix components (reducing their bioavailability), and/or the expression of specific genetic determinants of antibiotic resistance and tolerance (Hall and Mah, 2017). Since there was neither gross differences in matrix composition between the biofilms made by PAO1 and its isogenic *dnpA* insertion mutant nor changes in the susceptibility of the bacteria to antibiotics, we are left with the suggestion that, here also, the lower persister character of the mutant causes its higher susceptibility to fluoroquinolones in biofilms. Yet, the difference vanishes over time, possibly indicating a more preponderant role of the matrix barrier in more mature biofilms.

*DnpA* encodes a putative de-*N-*acetylase, the substrate of which is unknown. In other pathogens like *E. coli, S.* Typhimurium, or *Mycobacterium tuberculosis*, major signaling pathways involved in persistence are the toxin-antitoxin (TA) modules (Maisonneuve et al., 2011; Helaine et al., 2014). Acetylation-deacetylation reactions are central to the functioning of many of these modules. For example, acetylation of TacT toxin in *S.* Typhimurium blocks tRNA translation, arrests cell growth and increases persistence, while deacetylation detoxifies the cells, releasing the cells from their persister state (Cheverton et al., 2016). The role of TA modules in persistence has been proposed for *P. putida* (Tamman et al., 2014) but not described for *P. aeruginosa* so far. Yet, our data suggests that further investigation in this direction could be interesting. Moreover, acetylation and de-*N*-acetylation reactions are important for the architecture of the biofilm matrix. More specifically, acetylated alginate promotes bacterial aggregation while non-acetylated forms rather promote stigmergy (Fata Moradali et al., 2015). Partial acetylation is also described for Pel (Jennings et al., 2015) but not for PsI. The fact that *dnpA* inactivation does not affect biofilm formation suggests that neither alginate nor Pel are substrates for this enzyme, or, alternatively, that deacetylation reactions do not impact matrix properties since PsI is probably the main polysaccharide of the matrix for PAO1 (Ghafoor et al., 2011; Colvin et al., 2012) and is involved in the initial steps of attachment.

Importantly also, among the three classes of antibiotics tested, only fluoroquinolones showed improved activity against the *dnpA* insertion mutant. Although we do not have any molecular explanation for this specificity, we found that exposure of PAO1 to fluoroquinolones caused a marked upregulation of *gyrA* that was considerably reduced for the *dnpA* insertion mutant. Induction of *gyrA* expression has been observed in *E. coli* exposed to fluoroquinolones (Neumann and Quiñones, 1997), and may play a role in its tolerance to antibiotics. The decreased upregulation of *gyrA* in the *dnpA* insertion mutant might thus be a reason for its lowered tolerance toward fluoroquinolones compared to PAO1. It may also suggest a possible involvement of DnpA in DNA gyrase regulation. Noticeably, and in contrast to a previous work (Masuda et al., 2000), we also observed an induction of *mexX* expression by fluoroquinolones, but which was lower in the mutant. While no relation between the expression level of *gyrA* and *mexX* has been established, an overexpression of *mexX* has been described in fluoroquinolone-resistant *gyrA* mutants (Niga et al., 2005).

Our study suffers from two main limitations. First, we could not elucidate the enzymatic function of DnpA, neither its specific role in persistence. This would require transcriptomic or metabolomic comparison of the mutant and wild-type strains that were out of the scope of this pharmacologically oriented study. Second, we did not study the expression of *dnpA* in phagocytosed bacteria or in biofilms. Yet, overexpression of DnpA in PAO1 does not confer any phenotype, suggesting that it is the presence of the DnpA protein rather than its expression level which contributes to persistence. In spite of these limitations, our work clearly demonstrates that this poorly characterized de-*N*-acetylase can severely alter the response of *P. aeruginosa* toward fluoroquinolones in models relevant for persistent infections. It also highlights, for the first time, a possible link between de-*N*-acetylation and the regulation of topoisomerase expression. In a pharmacological perspective, this study may open the door to the search of anti-persister strategies.

## Author Contributions

SK, MF, JM, and FVB conceived and designed the experiments. SK performed the experiments. VL constructed part of the strains. SK, MF, PT, JM, and FVB analyzed the data. SK and FVB wrote the paper, which was reviewed by all co-authors.

## Conflict of Interest Statement

The authors declare that the research was conducted in the absence of any commercial or financial relationships that could be construed as a potential conflict of interest.
