# Supplementary material for: The Putative De-N-acetylase DnpA Contributes to Intracellular and Biofilm-Associated Persistence of Pseudomonas aeruginosa Exposed to Fluoroquinolones
Source: Front Microbiol. 2018 Jul 10;9:1455. doi: 10.3389/fmicb.2018.01455 (PMC6048251; doi:10.3389/fmicb.2018.01455)
Supplement: Supplementary file 1 [file Data_Sheet_1.pdf]

Supplementary Material

The putative de-*N*-acetylase DnpA contributes to intracellular and biofilm-associated persistence of *Pseudomonas aeruginosa* exposed to fluoroquinolones.

Shaunak Khandekar, Veerle Liebens, Maarten Fauvart, Paul M. Tulkens, Jan Michiels, Françoise Van Bambeke*

*** Correspondence:** francoise.vanbambeke@uclouvain.be

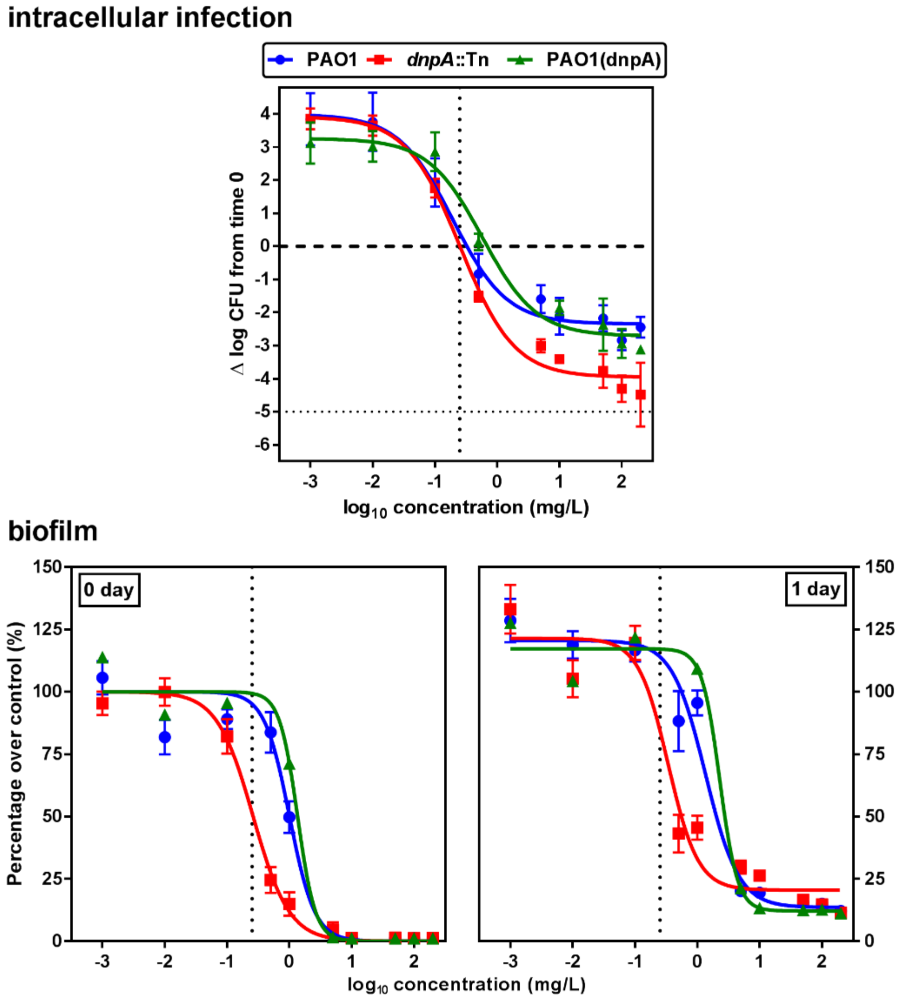

Figure S1: Comparison of the activity of ciprofloxacin against PAO1, its *dnpA* insertion mutant (*dnpA*::Tn), and PAO1 overexpressing *dnpA* (PAO1(dnpA); transformed by pME6032 plasmid with *dnpA* gene (PA5002), Tc^r)(Jacobs et al., 2003, Liebens et al., 2014).

Top: Concentration-response curves of ciprofloxacin against intracellular *P. aeruginosa*. The graphs show the changes in the number of cfu/mg of cell protein in THP-1 cells after 24 h of incubation with increasing extracellular antibiotic concentrations. Values are the mean ± SEM of at least 3 experiments performed in triplicates. Vertical dotted lines indicate MIC value in broth, the tick horizontal dotted line, a bacteriostatic effect, and the thin dotted line, the limit of detection.

Bottom: Concentration-response curves of the activity of ciprofloxacin against cell viability in 0 and 1 day-old biofilms of *P. aeruginosa*. The ordinate shows the percentage of residual fluorescein fluorescence signal as compared to control biofilms. The vertical dotted line indicates MIC value in broth. Values are the means ± SEM of at least 3 experiments performed in triplicates.

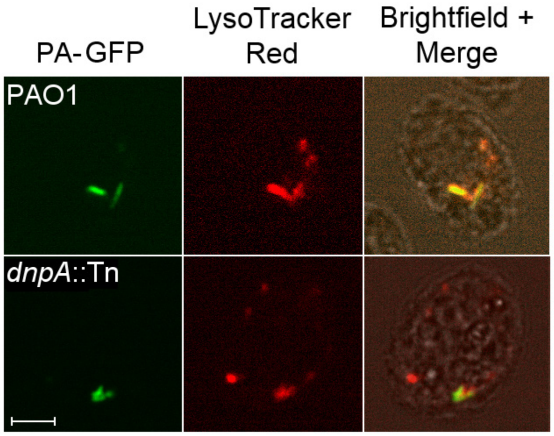

Figure S2: Confocal microscopy images of THP-1 monocytes infected by PAO1(GFP) (top) or – *dnpA*::Tn(GFP) (bottom) after 24 h incubation with 50x MIC of ciprofloxacin. Acidic bodies were stained with LysoTracker Red. Bar: 5 µm.

For this experiment; intracellular infection was performed as described in the article, using PAO1(GFP-iptg) and *dnpA*::Tn(GFP-iptg). Infected cells were incubated with 50x MIC of antibiotics during 24h, collected by centrifugation (900 rpm, 7 min), resuspended in pre-warmed RPMI-1640 containing 50 nM LysoTracker Red (Life Technologies, Carlsbad, CA), incubated at 37°C for 45 min, centrifuged again, resuspended in half the volume of RPMI-1640 containing 10% foetal calf serum and loaded on fibronectin coated µ-slide (Ibidi, Martinsried, Germany). After 15 min cells were observed in Cell Observer SD confocal microscope (Carl Zeiss AG, Oberkochen, Germany) using spinning disc technology (Yokogawa, Tokyo, Japan) and controlled by the AxioVision software (AxioVs40 version 4.8.2.0). Excitation and emission wavelengths were set at 488 nm and 520/35 nm (GFP) and (635 nm and 685/40 nm (Lysotracker-Red), respectively.

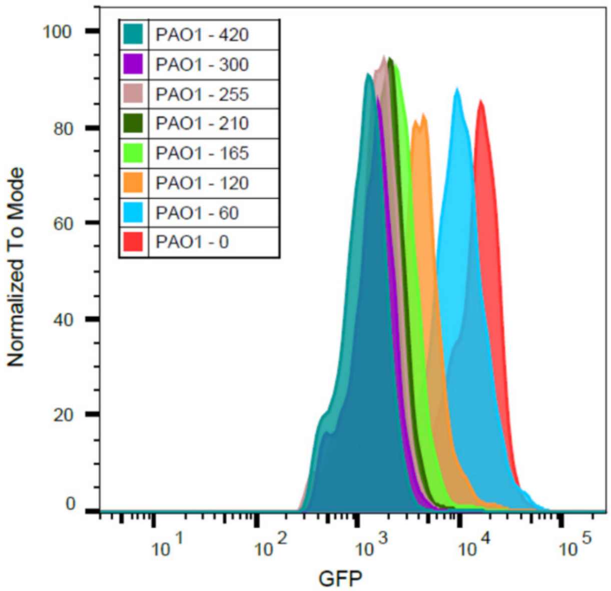

Figure S3: Fluorescence dilution experiment performed on planktonic *P. aeruginosa* PAO1 harbouring GFP. The histograms show the number of events (normalized to the median value) as a function of the intensity of GFP signal; a shift of histograms over time to the left indicates a reduction in fluorescence indicative of bacterial multiplication. The numbers in the legend indicate the times (in minutes) at which the samples were harvested during bacterial growth. At least 10,000 events were measured for each sample.

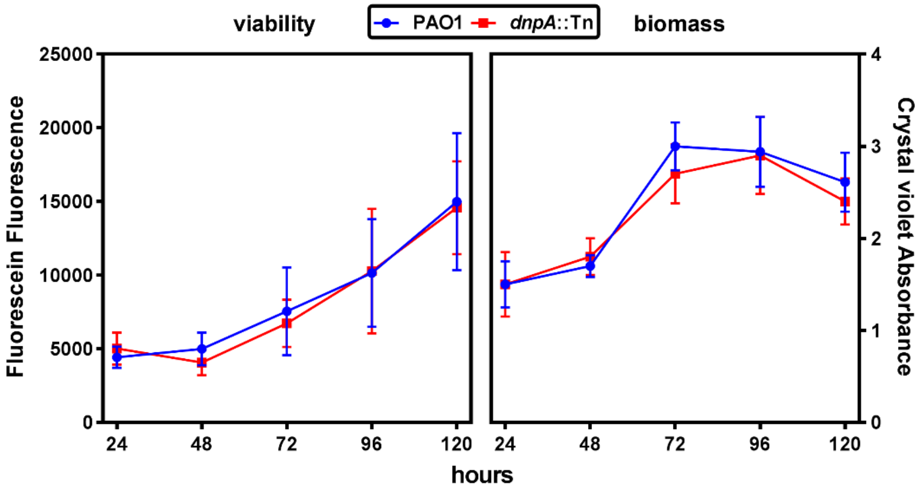

Figure S4: Kinetics of biofilm development by PAO1 and its *dnpA* deletion mutant (*dnpA*::Tn) in MHB-CA. Biofilms were gown in 96-well plates will daily replacement of the medium. Every day, 8 wells were harvested for measure of viability (assessed by the metabolic conversion of fluorescein diacetate in fluorescein; left graph) and 8 wells for measure of biomass (assessed by the absorbance of crystal violet; right graph). Data are the mean ± SEM of three independent experiments (n=8).

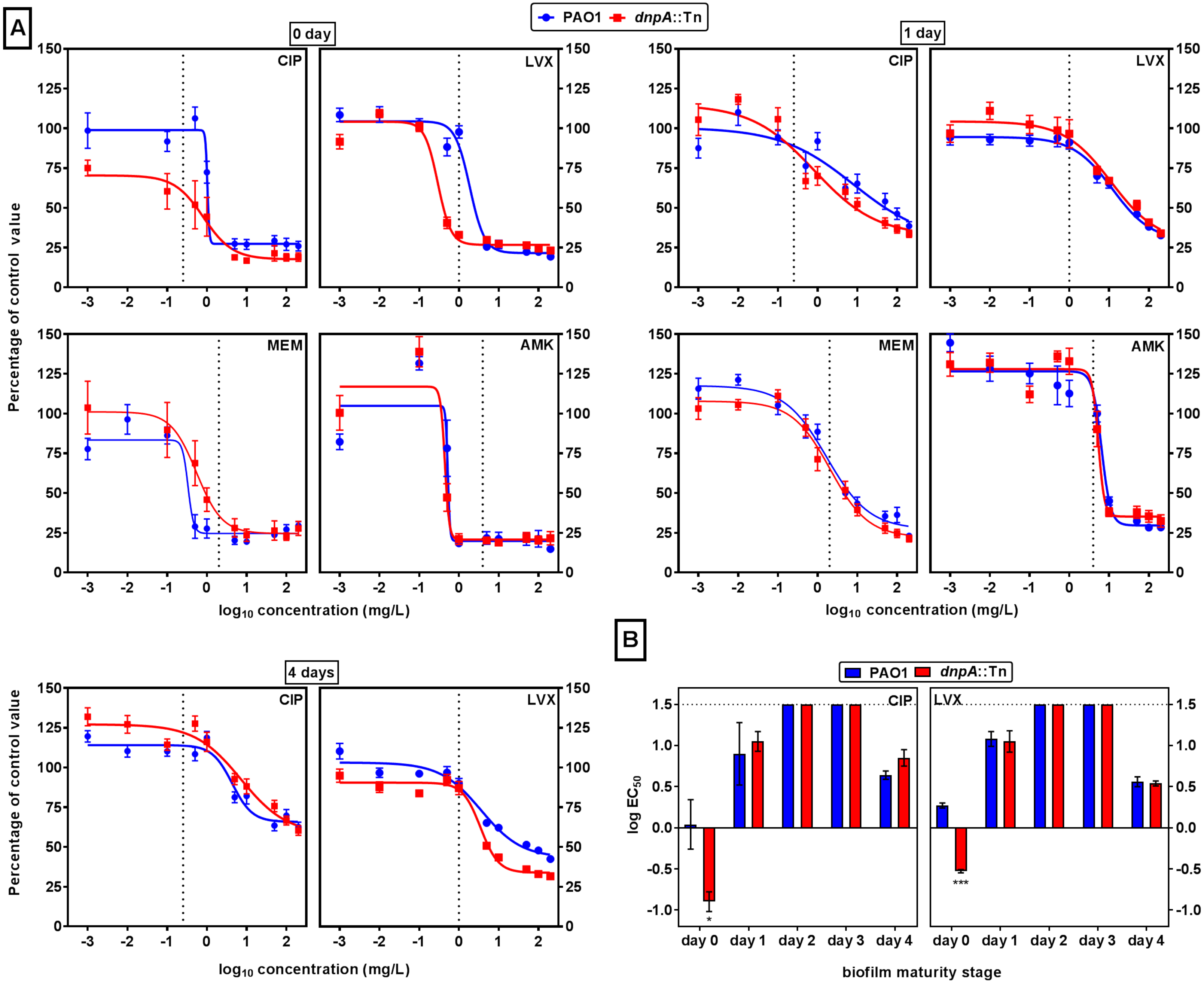
A
PAO1
dnpA::Tn
0 day
1 day
4 days
CIP
LVX
MEM
AMK
Percentage of control value
log10 concentration (mg/L)
B
log EC50
biofilm maturity stage
day 0
day 1
day 2
day 3
day 4
*
***

Figure S5: A. Concentration-response curve of the activity of antibiotics against biofilm biomass in 0, 1 or 4 days-old biofilms of *P. aeruginosa* strains. The ordinate shows the percentage of residual crystal violet absorbance signal as compared to control biofilms. The vertical dotted line indicates MIC value in broth. CIP, ciprofloxacin; LVX, levofloxacin; MEM, meropenem; AMK, amikacin. B. Comparison of the $logEC_{50}$ (calculated from the Hill equation of the concentration-response curve presented in panel A or of similar experiments at days 2 or 3) as a measure of the relative potency of fluoroquinolones against biomass. Values > 1.5 were not calculated (out of the range investigated) and set arbitrarily to 1.5. Values are the means ± SEM of at least 3 experiments performed in triplicates. Statistical analysis (multiple t-test) comparing *dnpA*::Tn to PAO1: *$p<0.05$, ***$p<0.001$.

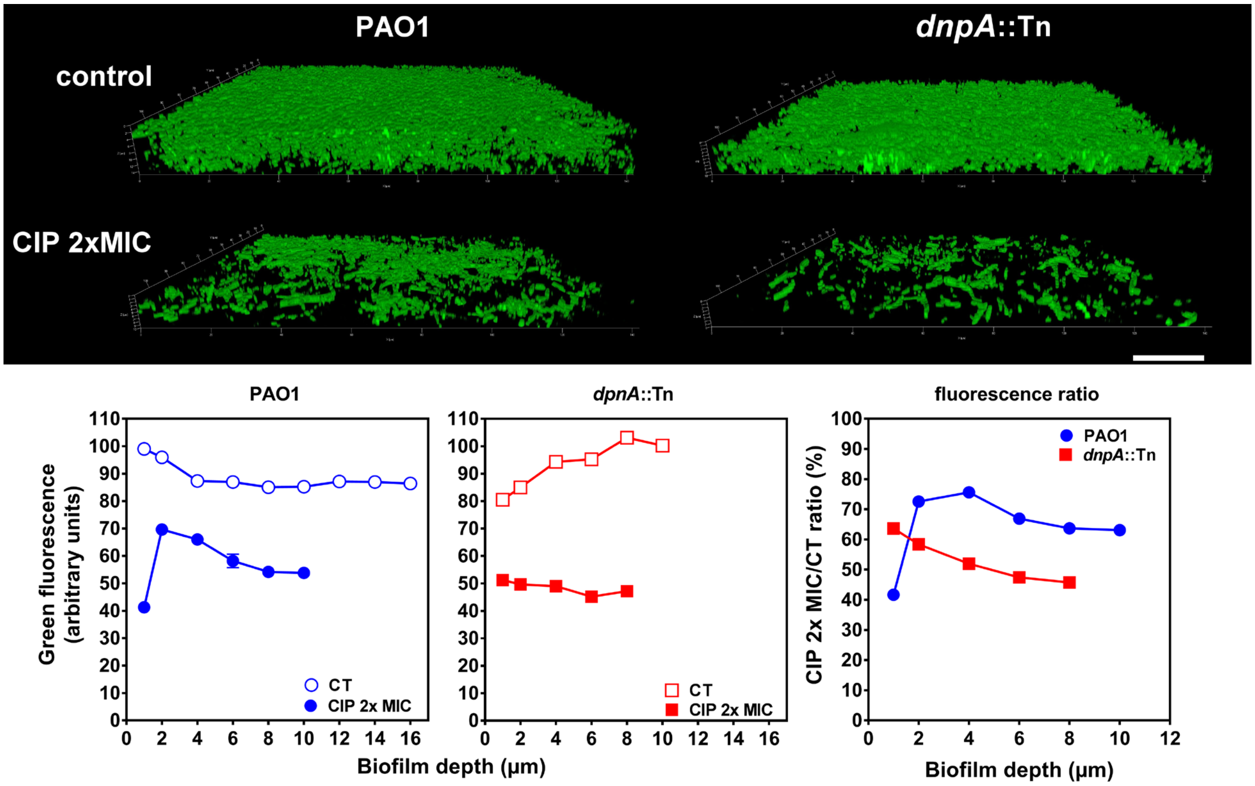

Figure S6: Top panel: Appearance in confocal microscopy of 1-day old biofilms from PAO1 (left) or the *dnpA* insertion mutant (right) that were incubated in control conditions or with ciprofloxacin at 2x MIC during 24 additional hours. Bacteria expressing GFP (green) were used for this experiment. Scale bar: 20 µm. CIP, ciprofloxacin.

Bottom panel: quantification of the fluorescence signal in biofilms illustrated in the top panel. The left and middle graph show the signal measured in the depth of the biofilm for PAO1 and the *dnpA* insertion mutant respectively. The right panel shows the ratio between the fluorescence signal measured in the presence of ciprofloxacin and in control conditions for the two strains

The thickness of the biofilm formed by *dnpA*::Tn seemed slightly lower than that of the biofilm formed by PAO1, but the difference was not significant (*dnpA*::Tn: 11.2 ± 2.8 µm; PAO1: 14.2 ± 1.7 µm; p= 0.07 based on 5 independent experiments). The quantitative analysis of the fluorescence signal presented in the lower panel of the figure shows a reduction to 60-45% of control values in the presence of ciprofloxacin at 2x MIC in the *dnpA*::Tn mutant against a reduction to only 75-63% of control values in PAO1. Note however, that the support used to grow biofilms (glass in confocal microscopy *vs*. plastic for quantitative measures) could influence biofilm production, so that comparing the quantitative data presented in Figure 5 of the paper with what can be grasped from the confocal images needs to be made with caution.

Method for this experiment:

PAO1 and *dnpA*::Tn harboring PBBR5(GFP) were used. 1 mM IPTG was added to the medium for all the steps (GFP expression is constitutive so this addition is not expected to affect GFP expression). Biofilms were grown on glass coverslips (18 mm x 18 mm; VWR), which were placed in 6-well plates, covered with 2 mL of bacterial suspension, and incubated at 37°C for 1 day. Antibiotics were added at 2x or 50x MIC for 24 h. After 24 h, the medium was aspirated from the wells. The coverslips were then put on glass slides and samples were imaged with a Cell Observer SD confocal microscope (Carl Zeiss AG, Oberkochen, Germany) using spinning disc technology (Yokogawa, Tokyo, Japan), controlled by the AxioVision software (AxioVs40 version 4.8.2.0, Carl Zeiss GmbH, Jena, Germany) and with excitation and emission wavelengths set at 488 nm and 520/35 nm. Image stacks were acquired and recorded using a z-stack module and used to construct three-dimensional images.

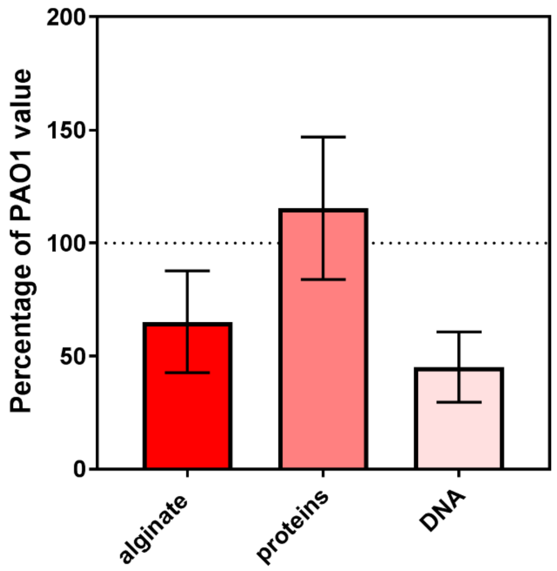

Figure S7: Analysis of biofilm exopolymers, with quantification of DNA, proteins and alginate. Data are expressed as the ratio between the amounts of material measured in *dnpA*::Tn *vs*. PAO1. Error bars represent SEM (minimum 3 experiments performed in triplicates). Statistical analysis (multiple t-test) comparing *dnpA*::Tn to PAO1: no statistical differences

For this experiment, biofilms were grown as for confocal imaging in 6-well plates. Matrix constituents were assayed using procedures similar or adapted from published methods. DNA was measured exactly as described (Siala et al., 2016). Proteins were precipitated (Foulston et al., 2014), washed with 1 mL ice-cold acetone, air dried at room temperature, resuspended in 1 mL PBS and measured using the DC Protein Assay kit (Bio-Rad) following manufacturer's instructions. For exopolysaccharides, biofilms were centrifuged at 3,500 g for 20 min; pellets were resuspended in PBS, and mildly sonicated for 5 min in a sonicator bath. After centrifugation, the supernatant was harvested and mixed with ice-cold 100% isopropanol in a 5:1 ratio and incubated overnight at 4°C. Samples were again centrifuged, pellets resuspended in a digestion mix of 0.1 M $MgCl_2$, 0.1 mg/mL DNase, and 0.1 mg/mL RNase solution, mildly sonicated and incubated for 4 hrs at 37°C. Samples were then extracted twice with phenol/chloroform/isoamyl alcohol (25:24:1) (Elsholz et al., 2014). The top aqueous fraction was collected and dialyzed for 48 hrs with 12kDa cut-off membrane (Sigma-Aldrich). A standard dose response curve was built using sodium alginate (Sigma-Aldrich) as reference (0.25-20 mg/mL). Each standard was thoroughly mixed with 2-fold excess of 3 M sulfuric acid and incubated at room temperature for 5 min before reading of absorbance at 360 nm. The biofilm samples were treated and analysed using the same procedure.

References

Elsholz AK, Wacker SA, Losick R. Self-regulation of exopolysaccharide production in Bacillus subtilis by a tyrosine kinase. *Genes Dev* (2014) 28(15):1710-20. doi: 10.1101/gad.246397.114.

Foulston L, Elsholz AKW, DeFrancesco AS, Losick R. The Extracellular Matrix of Staphylococcus aureus Biofilms Comprises Cytoplasmic Proteins That Associate with the Cell Surface in Response to Decreasing pH. *mBio* (2014) 5(5). doi: 10.1128/mBio.01667-14.

Jacobs MA, Alwood A, Thaipisuttikul I, Spencer D, Haugen E, Ernst S, et al. Comprehensive transposon mutant library of Pseudomonas aeruginosa. *Proc Natl Acad Sci U S A* (2003) 100(24):14339-44. doi: 10.1073/pnas.2036282100.

Liebens V, Defraine V, Van der Leyden A, De Groote VN, Fierro C, Beullens S, et al. A putative de-N-acetylase of the PIG-L superfamily affects fluoroquinolone tolerance in Pseudomonas aeruginosa. *Pathog Dis* (2014) 71(1):39-54. doi: 10.1111/2049-632X.12174.

Siala W, Kucharíková S, Braem A, Vleugels J, Tulkens PM, Mingeot-Leclercq M-P, et al. The antifungal caspofungin increases fluoroquinolone activity against Staphylococcus aureus biofilms by inhibiting N-acetylglucosamine transferase. *Nat Commun* (2016) 7. doi: 10.1038/ncomms13286.
